# News and Events of the Week

**Published:** 1910-01-08

**Authors:** 


### News and Events of the Week.

There was issued on January 1 the preamble and text of a Bill designed to empower the Society of Apothecaries to grant diplomas in Sanitary Science, Public Health, State Medicine, School Hygiene or Tropical Medicine, and in Dentistry or Dental Surgery, and for other purposes.

The Third International Congress of Physiotherapy will be held in Paris, March 29 to April 2, 1910, under the presidency of Professor Landouzy, dean of the faculty of medicine of Paris, and the secretaryship of M. Vaquez. An exposition of everything appertaining to physiotherapy will be held in conjunction with the Congress. Correspondence should be addressed to M. Vaquez, Secrétaire Général, 27 rue du Général-Foy, Paris.

The Norwegian barque *Romittent,* bound from Laguna to Havre, with a cargo of mahogany, was towed into Queenstown on Monday afternoon. The captain reported that the vessel was upwards of 100 days on the passage, during which the crew were nearly all striken by beri-beri. Two of the men died from the disease, and it was with the greatest difficulty that the vessel was navigated to the Irish coast. The vessel has been placed in the quarantine roads, and the port sanitary doctor has proceeded on board.

Dr. Henrich von Rankf, of Arcostrasse, Munich, Bavaria, a member of the Bavarian Privy Council, and a retired professor of the University of Munich, a vice-president of the Agricultural Society of Bavaria, formerly house surgeon to the German Hospital in London, afterwards in the service of the English Government in the Crimea during the Crimean war, and later in that of the Prussian Government during the Austro-Prussian war, who died on May 13, aged 78, left personal estate in the United Kingdom valued at £4,425.

A correspondent writes to the *Pharmaceutical Journal* : Can you inform me what butchers in various parts of the country supply as "sweetbreads"? Dictionaries, text-books of anatomy, etc., give "Sweetbread—the pancreas." A sweetbread was ordered for a friend of mine, and the butcher was casually asked where it was situated. He said "in the throat," and the gland supplied was undoubtedly the thyroid. Subsequent questions brought to light the fact that in butcher's language there are three "breads" —"throat-bread," "heart-bread," and "gut-bread." The first is evidently the thyroid, and the last the pancreas; whether the second is the thymus I cannot at present say. But I was informed that the gut-bread, *i.e.* the sweetbread of dictionaries and doctors, is "given to paupers or thrown away," while the throat-bread is the luxury of the rich. When doctors order sweetbreads for invalids, do they intend the pancreas, and do they usually get the pancreas?

To deal with the epidemic of typhoid at Montreal, a temporary hospital has been opened in a disused factory, which will give accommodation for 150 patients. Merchants are giving the requisite furnishings, and artizans of all trades have voluntarily contributed the necessary labour. The emergency hospital will be governed by a committee of prominent citizens, headed by Sir Edward Clouston and Colonel Burland. The medical and nursing professions have responded nobly to the call for assistance. The activity of all these distressed persons is in striking contrast with the indifference of the City Council. Archbishop Bruchesi has announced that if the hospitals are unable to accommodate the fever patients he will open the convents and religious institutions of the archdiocese for their benefit. There have been 34 deaths in three days.

A striking instance, not without humour, of the use of statistical literature by those with whom such literature concerns itself is given in the *Daily Mail* of January 1 :—"A blind man, William Henry Rooke, a vendor of literature at street-corner meetings in South London, went into the witness box at Tower Bridge Police Court (on December 31) carrying Burdett's *Hospitals and Charities, 1905: The Year-Book of Philanthropy and Hospital Annual.* He said that 'he found by *Burdett's Annual* that thousands of pounds were contributed for the relief of necessitous people, but they got very little of it, the greater proportion being spent in large salaries for officials.'—Mr. Rose : Have you tried to get relief from a society for the blind?—The applicant said that he had tried several times and had succeeded in getting only a little. He added that salaries as high as £1,000 were paid, while salaries of £300 seemed to be paid to clerks. But, he went on, you can always get a decent clerk for £100 a year. I think that out of every 10s. contributed by the philanthropic only 4s. goes to blind people and 6s. to the expenses." Whether we agree with Mr. Rooke's opinions or not, we are extremely glad to find any reader of his necessarily busy class studying statistics as intelligently as he has done.

At Fulham Coroner's Court on Tuesday an inquest was held on the body of a woman who died from strychnine poisoning. Her husband stated that his wife had complained of internal pains; on Friday night his wife, who had sent for Dr. Fletcher during the day, said to him, "That medicine is burning my inside out. I am dying." The witness sent for Dr. Fletcher, who immediately used the stomach pump and a large quantity of water. Dr. John Fletcher said the deceased told him she was suffering from certain internal symptoms, and he went to the surgery to get some opium and chloroform water to give her. About half an hour afterwards he was called to the house and was told the woman was in a fit. He immediately used a stomach pump. It suddenly flashed across his mind that the chloroform bottle and the strychnine bottle were about the same size. Pathological evidence showed that the death was due to strychnine poisoning. The Coroner said that in the whole of his experience he did not remember having had a more painful case. It was a terrible mistake to make. It was due to the frankness of Dr. Fletcher, however, that they could come to a decision as to the cause of death without an analysis. The jury returned a verdict of "Death from misadventure," and asked the Coroner to bring to the notice of the Home Office the facts of the case, in order that legislation providing for a distinctive form of bottle for poison might be brought about.

It is satisfactory to learn that in all the steamers which the Orient Line possesses the open steerage berth accommodation has been now abolished. This means that every passenger on the Orient boats going to Australia third-class will yet enjoy the privacy of an enclosed cabin. In the five new vessels of 12,000 tons recently acquired by the company the comfort of the third-class passenger has been carefully looked after.

